# Diversity, Equity, and Inclusion in Action: Planning, Leadership, and Programming

**DOI:** 10.29173/jchla29608

**Published:** 2022-04-01

**Authors:** Andrew Barber

**Affiliations:** Library and Information Science Circulation Supervisor, Prescott College, Prescott, Arizona USA

Bombaro C. **Diversity, Equity, and Inclusion in Action: Planning, Leadership, and Programming**. Chicago: ALA Editions; 2020. Paperback: 208 p. ISBN: 978-0-8389-4759-3. Price: USD$67.99. Available from: https://www.alastore.ala.org/content/diversity-equity-and-inclusion-action-planning-leadership-and-programming

Adopting a commitment to diversity, equality, and/or inclusivity has grown in popularity across America’s higher education institutions and their libraries. These commitments can take on many forms in academic libraries ranging from a simple statement of non-discrimination to actionable plans with funding for programming, trainings, collection development, and beyond. However, to say that we as library professionals work in a perfect state of equality across ability, race, gender, sexual orientation, age, or economic class would be a lie.

*Diversity, Equity, and Inclusion in Action: Planning, Leadership, and Programming* is a collection of chapters that detail a wide range of diversity, equity, and inclusion (DEI) programs from librarians at American academic libraries. Many of the authors included in this collection critique current and historical efforts in librarianship to tackle social inequities, in theory and in practice, while sharing ideas, frameworks, or case studies of their own work.

The book is edited by Christine Bombaro, Associate Director for Research and Instructional Services at Dickinson College. In their introduction, Bombaro accurately depicts the state of DEI and social justice work in librarianship as a challenging and slow process that has thus far fallen short of reaching a true state of social justice in the profession. From the chapters compiled in this collection, Bombaro hopes that readers can 1) critically assess the state of social justice work at their institutions and 2) become inspired by others already doing this work to take up the baton and continue the tradition of the librarian activist in their own communities. This collection strives to present readers with actionable solutions and DEI initiatives, but given the nature and difficulty of the work as acknowledged in the introduction, falls short of achieving that.

Although the book appears to be an introductory resource for libraries considering implementing DEI initiatives in their workplaces, readers will benefit from previous exposure to social justice literature and concepts before picking up this volume. While the language presented here is far more accessible than the complex jargon of critical theorists, it is generally assumed that the reader understands racism, sexism, and homophobia as systems of oppression deeply embedded in American society. For example, while “social justice” is defined in the first chapter, the term is used loosely and interchangeably across the selected essays adding to possible confusion for readers not familiar with DEI terminology and diluting the overall message.

The authors collected here organized, and in some instances facilitated, projects with the intent of critically framing an issue of social equity pertinent to either their community or area of practice. Of note is a project at Jefferson College in Missouri where librarians were able to assist in forming an LGBTQ+ student organization on a campus where the LGBTQ+ student population was largely invisible. Kohlburn and Gomillion identified a need on their campus and consulted with students to amplify their voices. The librarians leveraged their access and familiarity with the college’s bureaucracy to not only help found the group but to continually support the student members for years to come with funding, visibility, administrative buy-in, and advocacy. These authors demonstrate that this kind of work requires persistent effort over many years to have a meaningful impact.

Other chapters present frameworks and curriculums for programs, trainings, or changes to practice that do not include writing on attempts at implementation. For example, in chapter 5 Higgins and Stark present a curriculum for identifying and mitigating implicit racial bias in health sciences research and instruction. While not a novel concept, the framework could prove useful to librarians looking for some guidance on adopting a critical approach to information literacy pedagogy.

The true merit of the collection lies in its discussion and strategies for working with leadership and securing funding for projects. Many of the authors here share their experiences working with provosts, deans, and vice presidents at their institutions including challenges and successes. The reader could cite the successes of the initiatives here as evidence to support investing in actionable programs and curriculum at their own libraries.

Unfortunately, like a substantial portion of DEI work, this collection fails to include a meaningful discussion of disability. When brought up, only visible, physical disabilities are mentioned in passing in relation to accessible furniture such as adjustable-height desks. Mental illness, in particular, continues to become more prevalent in younger generations meaning an ever increasing number of patrons we currently and will serve in the academic library live with some form of disability. Health sciences libraries, in particular, can serve to educate the campus community and the profession on dis/ability and how this intersects with race, gender, and sexual orientation to form a nuanced matrix of oppression. Including discussions addressing disability in the collection would have greatly improved the book's impact and meaningfulness.

To conclude, the value in *Diversity, Equity, and Inclusion In Action* lies in the potential for inspiration, strategy for developing a plan to implement, and guidance in navigating the college bureaucracy to get support beyond the library for social justice initiatives. Any theory discussed, particularly on the inherent violence in library neutrality, has already been teased out elsewhere by critical librarians. Given that comparable work can be obtained at no cost on librarian Twitter pages and other blogs, the cost of the book (USD$70) is prohibitively expensive and potentially serves as a barrier for entry. However, if you require inspiration and your library will purchase it from their professional development or collections budget, you might find something useful here.

